# Nanoscale Remodeling of Functional Synaptic Vesicle Pools in Hebbian Plasticity

**DOI:** 10.1016/j.celrep.2020.01.051

**Published:** 2020-02-11

**Authors:** Stephanie Rey, Vincenzo Marra, Catherine Smith, Kevin Staras

**Affiliations:** 1 Sussex Neuroscience, School of Life Sciences, University of Sussex, Brighton BN1 9QG, United Kingdom; 2 Department of Neuroscience, Psychology and Behaviour, University of Leicester, Leicester L1 7RH, United Kingdom

**Keywords:** presynaptic terminal, plasticity, long-term potentiation, synaptic vesicle, recycling pool, hippocampus, acute slice, electron microscopy

## Abstract

Vesicle pool properties are known determinants of synaptic efficacy, but their potential role as modifiable substrates in forms of Hebbian plasticity is still unclear. Here, we investigate this using a nanoscale readout of functionally recycled vesicles in natively wired hippocampal CA3→CA1 circuits undergoing long-term potentiation (LTP). We show that the total recycled vesicle pool is larger after plasticity induction, with the smallest terminals exhibiting the greatest relative expansion. Changes in the spatial organization of vesicles accompany potentiation including a specific increase in the number of recycled vesicles at the active zone, consistent with an ultrastructural remodeling component of synaptic strengthening. The cAMP-PKA pathway activator, forskolin, selectively mimics some features of LTP-driven changes, suggesting that distinct and independent modules of regulation accompany plasticity expression. Our findings provide evidence for a presynaptic locus of LTP encoded in the number and arrangement of functionally recycled vesicles, with relevance for models of long-term plasticity storage.

## Highlights

• Functional synaptic vesicle pools from hippocampus are analyzed ultrastructurally
• After a form of LTP induction, the average recycled pool fraction increases
• Spatial remodeling also occurs, with more recycled vesicles near the active zone
• The cAMP-PKA activator, forskolin, selectively mimics features of LTP remodeling

## Graphical Abstract

**Figure:**
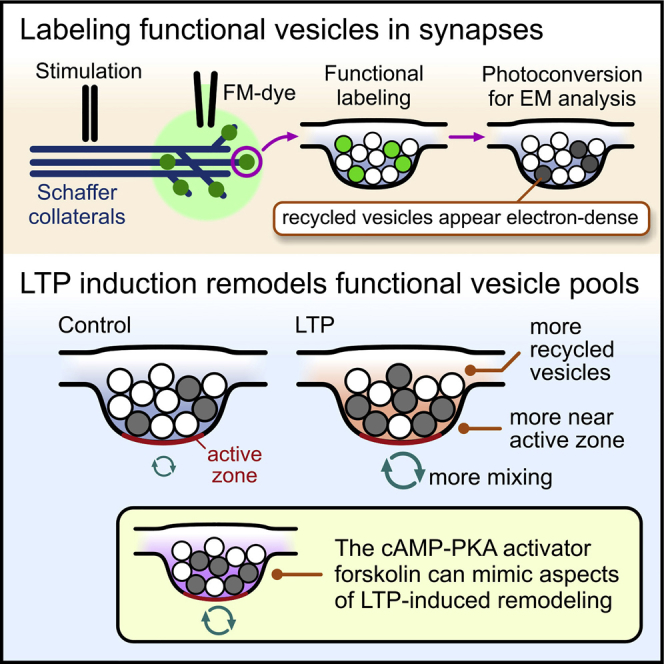

## Teaser

Vesicle pools are attractive potential substrates for encoding synaptic strength changes in plasticity. Using ultrastructural approaches, Rey et al. report that LTP induction remodels functional pools in CA3-CA1 hippocampal terminals, increasing the recycled vesicle fraction and positioning them relatively closer to the active zone.

## Introduction

Small central presynaptic terminals are characterized by a population of synaptic vesicles (SVs) that support regulated information signaling between neurons. Although SVs look morphologically equivalent, imaging and ultrastructural studies have demonstrated that the total vesicle population at a single terminal is sub-classifiable into functionally distinct pools (Betz and Bewick, 1992, Harata et al., 2001a, Rizzoli and Betz, 2004, Ryan and Smith, 1995, Schikorski and Stevens, 2001) including a recycling pool that readily undergoes activity-evoked turnover and a residual resting pool that is refractory to evoked release (Alabi and Tsien, 2012, Denker and Rizzoli, 2010, Fowler and Staras, 2015). Notably, characteristics of functional pools, including their size, spatial organization, and kinetics of use and retrieval, are known to correlate with measures of synaptic strength (Branco and Staras, 2009, Branco et al., 2008, Marra et al., 2012, Murthy et al., 1997, Park et al., 2012, Rey et al., 2015), lending weight to the idea that pools might be key substrates for enabling and storing changes in synaptic efficacy. Several lines of experimental evidence support this possibility. First, pool properties are highly variable across synaptic populations under basal conditions (Fernandez-Alfonso and Ryan, 2008, Fredj and Burrone, 2009, Harata et al., 2001b, Kim and Ryan, 2010, Li et al., 2005, Micheva and Smith, 2005, Ratnayaka et al., 2012, Rose et al., 2013, Welzel et al., 2011) and are modulated in a use-dependent manner (Kahms and Klingauf, 2018) and during developmental refinement (Rose et al., 2013). Second, changes in pool partitioning have been shown to accompany forms of homeostatic plasticity where the resting pool acts as a recruitable resource that supports enlargement of the recycling pool during chronic synaptic disuse (Kim and Ryan, 2010, Murthy et al., 1997, Thiagarajan et al., 2005). Third, elegant work has revealed key molecular pathways that contribute to the setting of recycling:resting pool fractions at single synapses and could thus account for regulated changes in pool segregation (Cazares et al., 2016, Guarnieri, 2017, Kim and Ryan, 2010, Marra et al., 2012, Ratnayaka et al., 2012).

If functional pool organization is a modifiable substrate for tuning synaptic efficacy, it raises the possibility that pools might be adjustable target substrates to encode and store sustained changes in presynaptic strength in forms of Hebbian potentiation, an idea with significance for models of long-term synaptic plasticity. This notion has support from a number of elegant studies based on fluorescence imaging approaches where changes in release kinetics of functional vesicle pools after long-term potentiation (LTP) induction have been reported (Bayazitov et al., 2007, Stanton et al., 2005, Tyler et al., 2006, Zakharenko et al., 2001).

Here, we set out to examine whether activity-recycled pools can be ultrastructurally remodeled after plasticity, a question demanding readouts of synaptic terminals to be made in natively wired circuits but with functional single-vesicle resolution and nanoscale context. We addressed this using a FM1-43 dye loading and photoconversion approach in acute hippocampal slices showing that under basal conditions, the pool of vesicles recycled by a saturating evoked stimulus in CA3→CA1 synaptic terminals represents a variable but, on average, small fraction of the total vesicle pool. After an LTP induction protocol, however, this mean recycled pool fraction approximately doubles in size, with the largest pool fractions observed in the smaller synapses. We also find associated changes in the relative physical positions of vesicle pools in the terminal volume, including alterations in the vesicle pool composition close to the active zone (AZ). Characteristics of this ultrastructural remodeling are selectively mimicked by treatment with the cAMP-PKA pathway activator, forskolin, suggesting that distinct and independent components of pool regulation accompany plasticity expression. Our findings extend the emerging view that some forms of long-term potentiation can influence indices of presynaptic performance (Antonova et al., 2001, Bayazitov et al., 2007, Bell et al., 2014, Bourne et al., 2013, Emptage et al., 2003, Malgaroli et al., 1995, Micheva and Smith, 2005, Ninan et al., 2006, Ostroff et al., 2002, Padamsey et al., 2017, Ratnayaka et al., 2012, Ryan et al., 1996, Stanton et al., 2005, Tyler et al., 2006, Wang et al., 2005, Zakharenko et al., 2001, Zakharenko et al., 2003), here identifying the morphological remodeling of vesicle pools as a key event.

## Results

### LTP Influences Functionally Recycled Pool Size

**Figure 1:**
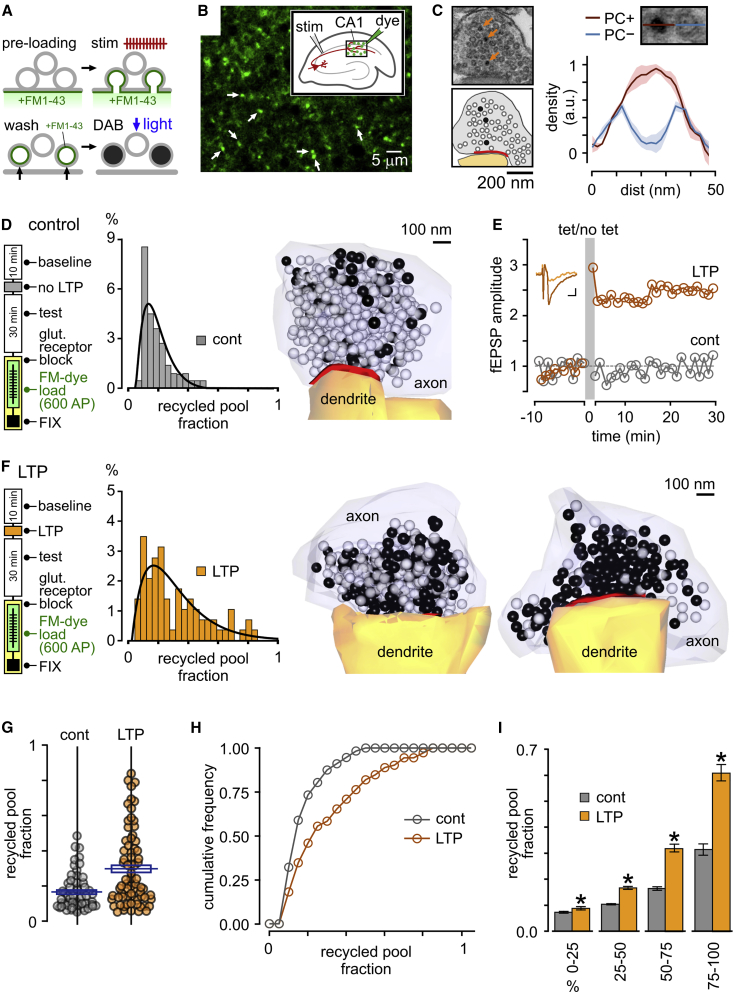
Expansion of the Functionally Recycled Pool with LTP (A) Cartoon schematic shows approach for ultrastructural readout based on functional FM dye loading. (B) Punctate FM1-43 dye labeling in CA1 region of hippocampus in response to stimulation of Schaffer collaterals (1,200-AP loading). (C) Electron micrograph shows activated synapse in target region characterized by vesicles with both electron-dense (arrows; red trace in profile plot on right is mean intensity ± SEM; n = 3 vesicles) and clear lumen (blue trace; n = 3 vesicles). (D) Control synapses dye labeled with a 600-AP stimulus and photoconverted for EM analysis. Left: protocol; for control, extracellular responses in CA1 to single electrical stimuli were recorded as “baseline” and “test” to match LTP protocol (see F). Slices were then washed into receptor blockers and FM1-43FX applied for 3 min before stimulation at 20 Hz for 30 s (600 APs) to dye label the recycled pool. Samples were microwave-fixed 3–4 min after the end of loading (“FIX”) and photoconverted for EM analysis (see STAR Methods). Middle: Distribution plot of recycled pool size (expressed as fraction of total pool) for control synapses (n = 56 synapses). Right: 3D reconstruction of typical control synapse labeled with a 600-AP stimulus. Dark spheres correspond to total recycling pool. Active zone is shown in red. (E) Plot of field EPSP (fEPSP) amplitude normalized to baseline for LTP and control protocols. Inset shows average fEPSP (5 traces) before and after LTP. Scale bars: 0.1 mV, 10 ms. (F) Left: protocol is same as control (D) with LTP induced electrically by three periods of tetanization (each 10 trains of 200-Hz stimulation delivered for 200 ms every 5 s) at 5-min intervals. (Middle) Distribution plot of recycled pool size for LTP synapses (n = 72 synapses). Right: 3D reconstructions of typical LTP synapses. (G) Scatterplot for control (n = 56 synapses) and LTP (n = 72 synapses) with blue horizontal lines in rectangle on each plot indicating mean and SEM, respectively. (H) Cumulative frequency distribution plot showing recycled pool fraction comparison for control (n = 56 synapses) and LTP (n = 72 synapses) synapses. (I) Histogram plot shows fractional pool sizes for control versus LTP (bars are ordered 25% bins of pool fractions of all synapses, mean ± SEM) with an increase in the size of high fractional pool sizes after LTP the most pronounced (right bars in plot; ^∗^p < 0.05, unpaired t tests). See also Videos S1 and S2.

To investigate plasticity-driven changes in SV pool organization, we used a combined functional and ultrastructural approach that allowed us to assay recycled vesicle characteristics directly at nanoscale resolution. Specifically, the activity-dependent optical marker FM1-43 (Betz and Bewick, 1992, Cousin et al., 2018, Gaffield and Betz, 2006, Ryan et al., 1993) (Figure 1A) was applied to CA1 in acute hippocampal slices during electrical stimulation of upstream Schaffer collaterals (Figure 1B, inset). This dye is internalized into recycling SVs and appears as punctate fluorescence in confocal images, consistent with functional synaptic labeling (Figure 1B, arrows) (Marra et al., 2012, Marra et al., 2014, Ratnayaka et al., 2011, Rey et al., 2015, Staras et al., 2010, Zakharenko et al., 2001). When photoactivated, this fluorescence can readily drive the polymerization of diaminobenzidine (DAB) to form an electron-dense osmiophilic precipitate in dye-filled vesicles in electron microscopy (EM) (Darcy et al., 2006, Denker et al., 2009, Denker et al., 2011, Harata et al., 2001b, Henkel et al., 1996, de Lange et al., 2003, Rizzoli and Betz, 2004, Schikorski and Stevens, 2001, Teng and Wilkinson, 2000) (Figures 1A and 1C).

We used this direct readout of recently retrieved SVs to characterize the ultrastructural properties of the total recycled pool recruited by a saturating loading stimulus at 20 Hz (600 action potentials [APs]). Quantifying the recycled pool fraction as the proportion of electron-dense (photoconverted [PC+]) vesicles in each photoconverted terminal revealed that synapses had highly variable fractional sizes with a small overall mean (0.16 ± 0.02, SD = 0.10, n = 56 synapses; Figures 1D and 1G; Video S1, related to Figure 1), consistent with our previous work (Marra et al., 2012) and that of others (Harata et al., 2001a, Harata et al., 2001b). We hypothesized that the activity-history experienced by individual synapses might be a factor that contributed to this population variability. To test this, we examined the effect of a global plasticity induction on synaptic pool properties by carrying out FM-dye acute-slice loading experiments that were time-matched with control experiments but preceded by an LTP-induction protocol already established to have a presynaptic component of expression (Bayazitov et al., 2007) (Figures 1E and 1F). In electron microscopy, we found that following this protocol, the mean recycled pool fraction in activated synapses was significantly higher than in controls (Figures 1F–1H; Video S2, related to Figure 1) with a broader spread of values (mean, 0.30 ± 0.03; n = 72 synapses; t test, p < 0.001; SD = 0.21; F test, p < 0.001; Figures 1F and 1G). Although control and LTP distributions have substantial overlap (compare distributions in Figure 1G), the higher overall mean fraction and broader spread in the LTP condition was principally driven by only a subset of synapses with very elevated recycled pool sizes (mean of top 25% synapses by fraction: control, 0.32 ± 0.02, n = 14 synapses; LTP, 0.61 ± 0.03, n = 18 synapses; t test, p < 0.001; Figure 1I).

Video S1. 3D Reconstruction of Typical Control Synapse Labeled with a 600 AP Stimulus, Related to Figure 1 Dark spheres correspond to total retrieved pool. Clear spheres are resting pool vesicles. Active zone is shown in red.

Video S2. 3D Reconstruction of Typical LTP Synapse Labeled with a 600 AP Stimulus, Related to Figure 1 Dark spheres correspond to total retrieved pool. Clear spheres are resting pool vesicles. Active zone is shown in red.

**Figure 2:**
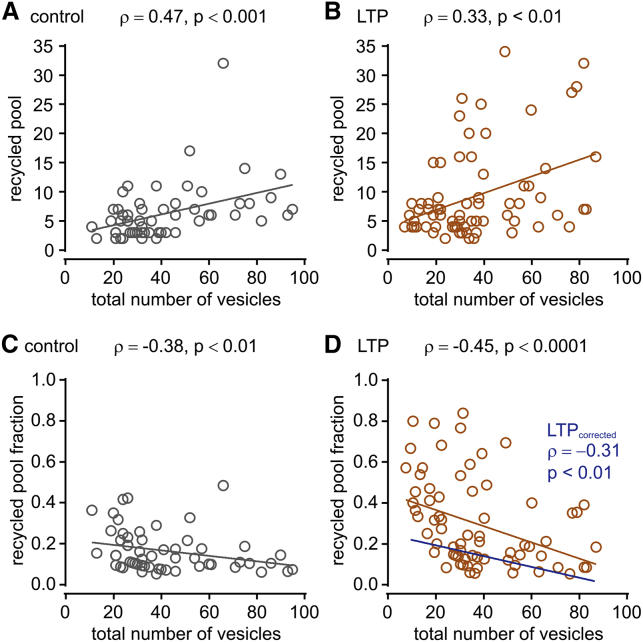
Pool Size Relationships in Control and LTP For all plots, numbers refer to Spearman’s correlation (ρ) and p values, respectively. (A and B) Scatterplot of recycled pool size versus total number of vesicles for control (A) (n = 56 synapses) and LTP (B) (n = 72 synapses) with linear fits. (C and D) Scatterplot of recycled pool fraction versus all vesicles for control (C) (n = 56 synapses) and LTP (D) (n = 72 synapses) with linear fits. Blue line (LTP_corrected_) in (D) shows fit after correcting for the expected pool size-pool fraction relationship observed in controls.

Notably, the recycled pool fraction was not expressed uniformly across all synapses. In both control and LTP synapses, there was a positive correlation between total vesicle number (synapse size) and recycled vesicle number (Spearman’s rank correlation, control: ρ = 0.47, p < 0.001, n = 56 synapses; LTP: ρ = 0.33, p < 0.01, n = 72 synapses; Figures 2A and 2B), and an inverse relationship between total vesicle number and recycled pool fraction (Spearman’s rank correlation, control: ρ = −0.38, p < 0.01, n = 56 synapses; LTP: ρ = −0.45, p < 0.0001, n = 72 synapses; Figures 2C and 2D) aligned with our previous work. Even after correcting for the pool size-pool fraction relationship observed in controls, a negative correlation between total vesicle number and recycled pool fraction persists in LTP (Spearman’s rank correlation, LTP_corrected_: ρ = −0.31, p < 0.01, n = 72 synapses; blue line in Figure 2D). In other words, synapses with the largest recycled pool fraction were typically smaller terminals, consistent with previous reports of synapse size dependency in plasticity (Bi and Poo, 1998, Malgaroli et al., 1995, Ratnayaka et al., 2012, Ryan et al., 1993). Collectively, our results demonstrate that a specific expansion of the recycled vesicle pool can accompany a form of long-term potentiation.

### Recycled Pool Organization Changes with LTP

**Figure 3:**
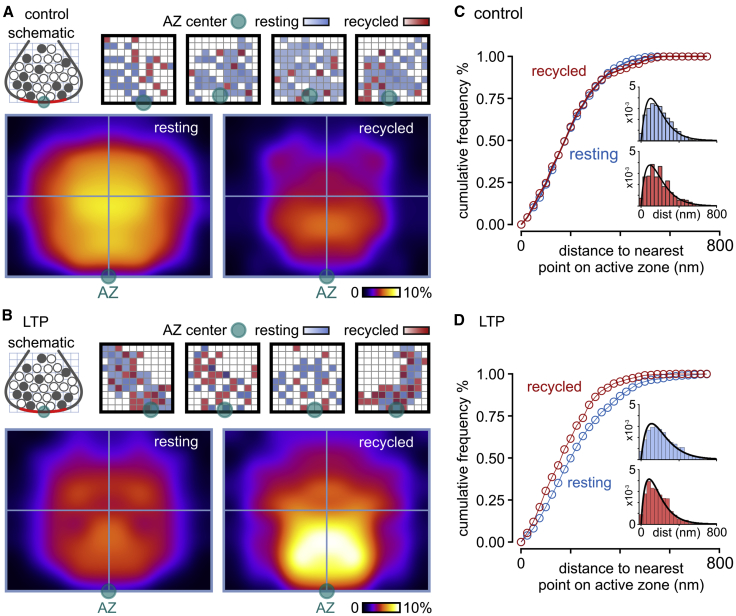
Retrieved Vesicles Occupy More Privileged Positions in the Synapse after LTP (A) Top left: schematic illustrates the cross-sectional view of vesicle pools depicted in all other panels with AZ center indicated by green-blue circle. Top right: density plots of the distribution of recycled (red) and resting (blue) vesicle pools for typical control synapses. Each grid square represents 1% of total vesicle cluster area with darker color shades corresponding to higher vesicle densities. Bottom: mean normalized and smoothed spatial frequency density plots for all synapses (n = 43 synapses) for resting and recycled vesicles. (B) As in (A) for LTP synapses (n = 52 synapses). (C and D) Cumulative frequency distribution plot for recycled (red) and resting vesicles (blue) for distance to nearest point on active zone for control (C) and LTP (D). Insets are distribution histograms of all vesicle distances for control (resting: n = 1,632 vesicles from 43 synapses; recycling: n = 278 vesicles from 43 synapses) and LTP (resting: n = 1,462 vesicles from 52 synapses; recycling: n = 522 vesicles from 52 synapses).

Next, we used the ultrastructural detail afforded by our method to investigate whether LTP influenced the physical positions of vesicles within the terminal architecture. Based on representative middle sections for each synapse, we mapped the coordinates of all recycled and resting vesicles with respect to the AZ and cluster boundaries and then collapsed these to generate mean spatial frequency distribution maps for each SV pool class across the synaptic population (Figures 3A and 3B). In control synapses, the organization of recycled and resting vesicles were broadly the same, with a density cloud centered around the cluster core (Figure 3A). However, in LTP synapses, the distribution maps were strikingly different, with recycled vesicles occupying sites closer to the active zone than resting ones (Figure 3B). This contrasting organization was confirmed by cumulative frequency plots based on linear distances between an individual vesicle and its nearest point on the active zone; in control synapses, recycled and resting vesicles had broadly overlapping distributions (Figure 3C), but in LTP terminals the resting pool distribution was comparatively right-shifted (Figure 3D). These findings are consistent with the idea that, following LTP, recycled vesicles replace resting vesicles in positions adjacent to the release site.

**Figure 4:**
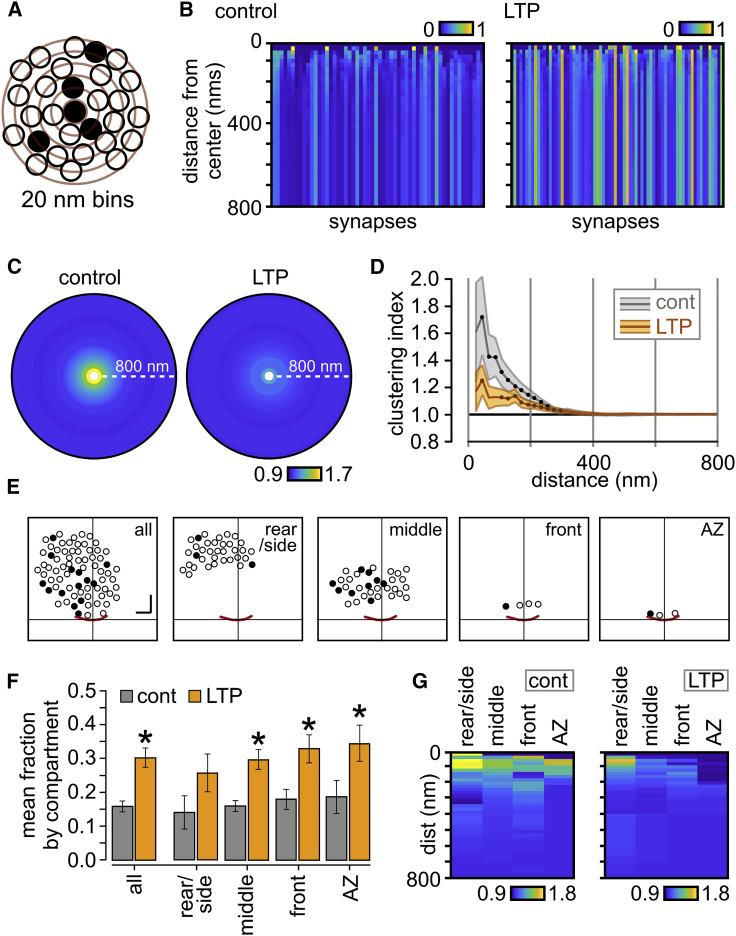
Increased Vesicle Intermixing after LTP (A) Schematic of cluster analysis approach. Recycling fractions are calculated for expanding concentric bins (20-nm intervals, red circles) around individual photoconverted (PC+) vesicles (filled circles). (B) Color-coded plots of mean absolute recycling fractions with distance away from PC+ vesicles; each synapse is represented by a vertical bar. Color scale indicates recycling fraction. (C) Mean circular frequency density plots showing relative PC+ clustering, normalized to final recycling fraction for the whole cluster (control, n = 56 synapses; LTP, n = 72 synapses). Color scale indicates clustering index. (D) Mean ± SEM plot of clustering index with increasing distance from vesicle center. Each filled circle indicates significant clustering (one-sample t tests versus 1, p < 0.01). (E) Cartoon illustrating compartment analysis used to examine regional differences in clustering, showing vesicles present in each compartment in an example synapse. Scale bars, 100 nm. (F) Histogram shows mean recycled fraction in each compartment. Values are significantly higher in compartments “all,” “middle,” “front,” and “AZ” in LTP versus control (two-way ANOVA, F(1,429) = 33.87, p < 0.0001, Bonferroni’s multiple-comparisons test for each compartment). (G) Plot showing relative PC+ clustering with increasing distance from PC+ vesicles for each compartment in control and LTP where color scale indicates clustering index.

We also examined how the recycled vesicles are positioned relative to each other by quantifying the recycled fraction in circular zones at increasing distances from each PC+ vesicle center (Figure 4A). Plotting the absolute fraction of PC+ vesicles as zone diameter increased, revealed that the LTP group had consistently larger recycled pool representation versus control (Figure 4B). Next, we calculated the size of the recycled pool fraction in the circular zones expressed relative to the final pool fraction for each synapse to provide a normalized measure of clustering, which was independent of the absolute recycled fraction. In both control and LTP synapses, PC+ vesicles exhibited significant local clustering (Figures 4C and 4D). We hypothesized that this might be principally driven by clustering in specific sub-compartments within the terminal. To test this idea, we divided the population of vesicles into distinct regions (“all,” “rear/side,” “middle,” “front,” and “active zone”; Figure 4E), defined by linear distances to the active zone, so that clustering properties could be examined independently in each compartment. Absolute recycled fractions were significantly higher in all, middle, front, and AZ compartments in LTP versus control (2-way ANOVA, F(1,425) = 32.08, p < 0.0001, Sidak’s multiple-comparisons test for each compartment for control versus LTP, comparison for rear/side, not significant) with the highest fractions seen as the distance to the AZ reduced (Figure 4F), consistent with our previous analysis (Figure 3). However, control terminals exhibited a trend toward higher peak clustering than LTP in all compartments (Figure 4G), suggesting a tendency for more vesicle intermixing with potentiation, although this difference was not significant (2-way ANOVA, F(1,248) = 2.49, p = 0.116). Likewise, there were no significance differences between compartments within control (1-way Kruskal-Wallis ANOVA, p = 0.94), or within LTP (1-way Kruskal-Wallis ANOVA, p = 0.70), suggesting that no single region drives the observed clustering.

### LTP-Driven Modulation of the Composition of the Active-Zone-Associated Pool

**Figure 5:**
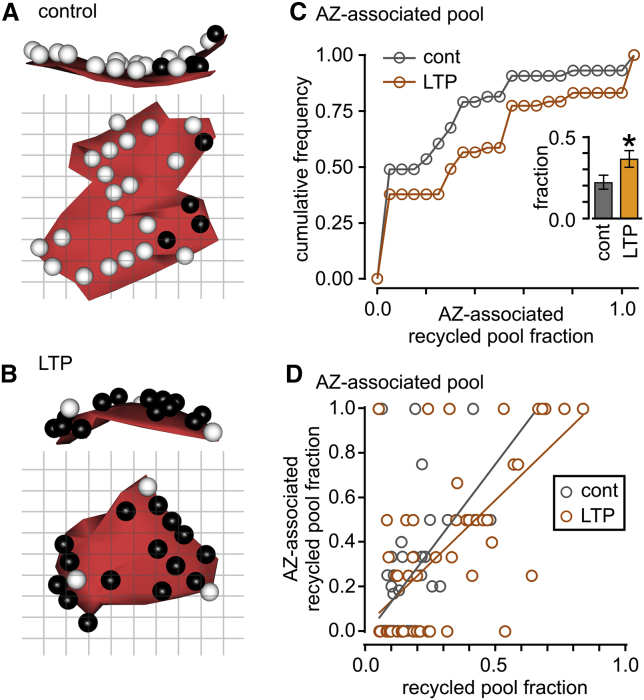
Increased Residency of Functionally Recycled Vesicles in the Active Zone-Associated Pool after LTP (A and B) Example 3-d reconstructions of active zones (az) for control (A) and LTP synapses (B). Dark spheres are recycled pool vesicles. Top panel: side view (cross-section); bottom panel: face view from terminal looking toward dendrite. (C) Cumulative frequency distribution plot showing recycled pool fraction in active zones for control (n = 43 synapses) and LTP synapses (n = 52 synapses). Inset shows mean recycled fraction ± SEM. (D) Scatterplot of the recycled fraction in the AZ-associated pool versus the recycled pool fraction of all vesicles for control (gray; n = 43 synapses; Spearman correlation ρ = 0.60, p < 0.001) and LTP (orange; n = 52 synapses; Spearman correlation ρ = 0.58, p < 0.001).

Next, we examined whether changes in the composition of recycled vesicle pools at the active zone itself might accompany LTP. We hypothesized that empty sites on the active zone, or those occupied by resting vesicles, could become functionalized by the insertion of releasable vesicles and therefore represent an important potential substrate for mediating synaptic strength changes (Pulido and Marty, 2017, Pulido et al., 2015). Consistent with previous observations revealing that the basal fraction of recycled vesicles near the active zone is low (Darcy et al., 2006, Harata et al., 2001b, Marra et al., 2012, Ratnayaka et al., 2012), we found that control synapses had a mean recycled pool fraction of just 0.22 ± 0.04 (mean ± SEM, n = 43 synapses). By contrast, synapses from the LTP condition showed significantly higher recycled vesicle pool occupation close to the active zone (0.36 ± 0.05, n = 52 synapses, t test, p < 0.028; Figures 5A–5C). As expected, we observed a significant correlation between the recycled fraction in the AZ and the total recycled fraction in both control and LTP (Figure 5D), consistent with the idea that these two pools approximately scale. To explore the increased presence of recycled vesicles near the AZ in LTP further, we looked at the composition of the AZ pool in “potentiated” synapses (defined here as the subset of synapses in the LTP group where the recycled pool fraction exceeded 0.36, two standard deviations above the mean fraction in control synapses). This strict definition included 34% of LTP synapses, and in these, recycled vesicles comprised nearly two-thirds of the AZ-associated pool (0.64 ± 0.08, n = 17 synapses) Moreover, the population of synapses with the top 25% of active zone recycled pool occupation had a mean fraction of 0.90 ± 0.05 (n = 13 synapses), suggesting that synapses can approach maximal filling of this vesicle population under potentiating conditions (Figure 5B). Our data provide ultrastructural evidence to show that an increased presence of functionally recycled vesicles associated with the AZ accompanies synaptic strengthening.

### High-Release Control Synapses Share Pool Organization Features with LTP Synapses

As a further type of comparison, we also examined how the population of synapses in the LTP group compared to the subset of synapses in the control group that had high recycled fractions; those that have a collective mean pool fraction that matched the mean pool fraction in the LTP group (control_high-fraction_ = 0.29 ± 0.03, n = 12, versus LTP_all_ = 0.30 ± 0.03, t test, p > 0.80, n.s.). We found that these two populations share other similarities; for example, they had comparable high fractions of recycled vesicles in the AZ-associated pool (control_high-fraction_, 0.42 ± 0.08, versus LTP_all_, 0.37 ± 0.05; t test, p > 0.54, n.s.) consistent with the idea that these parameters broadly scale (as in Figure 5D). Vesicle clustering properties in this subset of control synapses were intermediate between control and LTP (Figure S1A). However, other features observed in the LTP synapses were not apparent; for example, the closer proximity of recycled vesicles to the active zone, relative to the resting vesicle pool, is not seen in the control_high-fraction_ subset (Figure S1B). A speculative basis for these observations is that high-performing synapses in the control group might constitute those potentiated during their previous activity-history, and thus bear some persistent signatures of vesicle remodeling, comparable to features seen in our LTP synapses.

### Pharmacological Mimic of LTP-Driven Ultrastructural Changes

**Figure 6:**
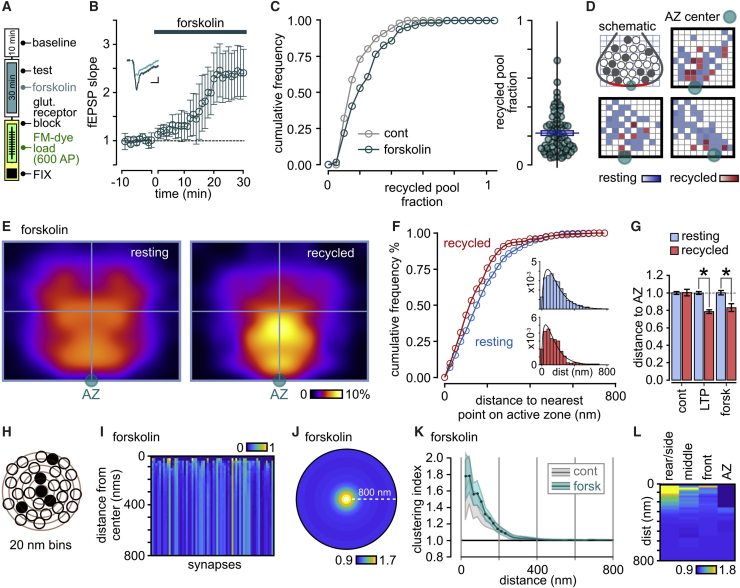
Forskolin Mimics Components of LTP-Driven Pool Remodeling (A) Labeling protocol. (B) fEPSP responses for 50 μM forskolin treatment (mean ± SEM; n = 3 slices). Inset shows fEPSP traces immediately before (light color) and 30 min after forskolin treatment (dark color). Scale bars: 0.2 mV, 5 ms. (C) Cumulative frequency distribution plot and scatterplot for forskolin treatment (n = 64 synapses). (D) Density plots of the distribution of recycled (red) and resting (blue) vesicle pools for typical synapses. Each grid square represents 1% of total vesicle cluster area with darker color shades corresponding to higher vesicle densities. The center of each active zone is indicated by a green-blue circle. Schematic illustrates the cross-sectional view of vesicle pools depicted in all other panels. (E) Mean normalized and smoothed spatial frequency density plots (n = 33 synapses) for resting and recycled vesicles. (F) Cumulative frequency distribution plot for recycled (red) and resting vesicles (blue) for distance to nearest point on active zone. Insets are distribution histograms of all vesicle distances (resting: n = 910 from 33 synapses; recycling: n = 248 from 33 synapses). (G) Histogram summarizing distance from each vesicle category to nearest point on AZ (resting and recycled counts for control: n = 1,632, 278 vesicles; LTP: n = 1,462, 522 vesicles; forskolin: n = 910, 248 vesicles; ^∗^ indicates significant comparisons, unpaired t tests). (H) Schematic of cluster analysis approach. Recycling fractions are calculated for expanding concentric bins (20-nm intervals; red circles) around individual photoconverted (PC+) vesicles (filled circles). (I) Color-coded plots of mean absolute recycling fractions with distance away from PC+ vesicles; each synapse is represented by a vertical bar. Color scale indicates recycling fraction. (J) Mean circular frequency density plot showing relative PC+ clustering, normalized to final recycling fraction for the whole cluster (n = 64 synapses). Color scale indicates clustering index. (K) Mean ± SEM plot of clustering index with increasing distance from vesicle center. Each filled circle indicates significant clustering (one-sample t tests versus 1, p < 0.05). The gray line and shaded region show mean ± SEM plot of control synapse for comparison. (L) Plot showing relative PC+ clustering with increasing distance from PC+ vesicles for each compartment in forskolin-treated synapses where color scale indicates clustering index.

cAMP-PKA pathway activation can drive increases in synaptic efficacy through actions on vesicle pools (Midorikawa and Sakaba, 2017, Sakaba and Neher, 2001, Yao and Sakaba, 2010) and mimic characteristics of synaptic strengthening observed in forms of electrically evoked LTP (Bayazitov et al., 2007, Castillo et al., 1997, Castillo et al., 2002, Lonart et al., 2003). Moreover, activation of this pathway expands the recycling pool in cultured hippocampal neurons (Willeumier et al., 2006). Importantly, PKA targets include structural and active zone proteins such as RIM1a (Castillo et al., 2002, Lonart et al., 2003) and SNAP-25 (Nagy et al., 2004), raising the possibility that structural remodeling of functional vesicle pools could contribute to presynaptic strengthening effects, analogous to our LTP findings. To test this hypothesis, we bath applied the adenylyl cyclase activator forskolin (30 min) to potentiate synapses (Figure 6B), and then labeled the total recycled pool for ultrastructural investigation (Figure 6A). In EM, forskolin-treated synapses had a mean fraction of 0.22 ± 0.02, SD = 0.14 (n = 64 synapses from 3 slices; versus control, unpaired t test, p = 0.019; Figures 6B and 6C) and a AZ-associated pool fraction that was intermediate between control and LTP (0.31 ± 0.05), suggesting that PKA activation might be contributing to the overall increases in the functionally recycled fraction seen in our electrically evoked LTP protocol. Moreover, spatial analysis demonstrated that the mean distance between recycled vesicles and the active zone was significantly reduced versus resting vesicles (Figures 6D–6F), again aligned with our LTP findings (Figure 6G). However, local vesicle clustering was more pronounced in forskolin-treated synapses versus LTP, analogous to control synapses (Figures 6H–6K), and a region-specific cluster analysis suggested that this arose principally from aggregation of those vesicles in the rear/side compartments of the cluster (Figure 6L), although differences between compartments were not significantly different (1-way Kruskal-Wallis ANOVA, p = 0.081). Our results indicate that activation of the cAMP-PKA pathway selectively mimics specific components of the vesicle remodeling of recycled vesicle pools observed in LTP.

## Discussion

Here, we used a sensitive ultrastructure-function readout to examine characteristics of vesicle pools in acute brain slices. Our approach permitted a direct quantification of both a functionally recruited and non-recruited pool and with the ultrastructural context to assess organizational changes in vesicle properties. Our baseline measurements confirmed previous studies showing that the fraction of synaptic vesicles recycled by a saturating stimulus was highly variable across the synaptic population (Fernandez-Alfonso and Ryan, 2008, Fredj and Burrone, 2009, Harata et al., 2001b, Kim and Ryan, 2010, Li et al., 2005, Micheva and Smith, 2005, Ratnayaka et al., 2012, Rose et al., 2013, Welzel et al., 2011), consistent with the idea that pool size is not constant and immutable but rather a potential substrate for encoding changes in synaptic efficacy. We tested this idea in the context of Hebbian plasticity in natively wired acute brain slices using an established compound LTP-induction protocol (Bayazitov et al., 2007) where the presynaptic plasticity expression has already been exhaustively previously characterized (Bayazitov et al., 2007, Zakharenko et al., 2001, Zakharenko et al., 2003) and known to require postsynaptic AMPA receptor (AMPAR) and L-type voltage-gated calcium channel (VGCC) activation but only partial dependency on NMDA receptors (NMDARs). Here, we demonstrate that key changes in pool organization take place; specifically, an increase in the recycled pool fraction and changes in the distribution of vesicles in the terminal, with LTP favoring increased vesicle intermixing and the positioning of recycled vesicles at sites near the active zone. Our findings build on important previous studies that have established a morphological presynaptic basis for LTP (Bell et al., 2014, Bourne et al., 2013, Chéreau et al., 2017) and the idea that parameters of presynaptic real estate are important for synaptic strength determination (Holderith et al., 2012, Schweizer et al., 2012, Sheng et al., 2012), offering insight into the nanoscale remodeling of vesicle pools that can accompany Hebbian plasticity expression.

That potentiating stimuli can influence the number of presynaptic contact sites and their sizes and also influence the specific rates of vesicle release and turnover has been elegantly demonstrated in a range of landmark studies (Bayazitov et al., 2007, Micheva and Smith, 2005, Ninan and Arancio, 2004, Ninan et al., 2006, Ryan et al., 1996, Stanton et al., 2005, Tyler et al., 2006, Zakharenko et al., 2001). However, whether organizational characteristics of these functional pools could be modifiable storage substrates for synaptic strength determination has remained unclear, although there is some precedent for this idea in key previous studies. For example, a robust link between recycled pool size and release probability has been demonstrated (Murthy et al., 1997), suggesting that tuning of pool size could have significant functional consequences. Consistent with this, tour de force studies in primary cultured neurons have shown that resizing of functional pools is seen in forms of homeostatic modulation where chronic synaptic silencing drives functional pool enlargement to effect increases in transmission (Kim and Ryan, 2010, Murthy et al., 2001, Thiagarajan et al., 2005). In recent years, understanding of the molecular control mechanisms that could serve to regulate the partitioning of recycling and resting pools has also started to emerge, with studies implicating the balance of CDK5 and calcineurin activity (Kim and Ryan, 2010, Marra et al., 2012, Orenbuch et al., 2012, Ratnayaka et al., 2012) and vesicle-tethering complexes composed of tomosyn-I, synapsin, and Rab3-GTPase (Cazares et al., 2016, Guarnieri, 2017) as pivotal control points. Likewise, nanoscopic investigations have provided insights into the physical positioning of vesicles in terminals, showing that close apposition to the release site correlates with higher release probability (Marra et al., 2012, Park et al., 2012), supporting the view that ultrastructural re-organization of pools might be of functional significance.

The active zone is the physical access point for vesicles to undergo activity-dependent exocytosis. Nonetheless, the structure is not functionally homogeneous; recent work has demonstrated that it comprises multiple discrete release sites where vesicle fusion takes place (Maschi and Klyachko, 2017), presumably corresponding to the non-random clustering of Ca^2+^ channels (Holderith et al., 2012). It is also well-established that the composition of the active zone as a whole, is typically heavily biased toward non-recycled vesicles (Darcy et al., 2006, Fowler and Staras, 2015, Harata et al., 2001b, Marra et al., 2012, Ratnayaka et al., 2012). We hypothesized here that expanding the number of release-ready sites on the active zone at the expense of ones occupied by resting vesicles, could provide a possible means to mediate rapid adjustments in synaptic efficacy, an idea with support from models proposed by others (Pulido and Marty, 2017, Pulido et al., 2015). Elegant high-pressure freezing EM approaches suggest that interactions between vesicles and the active zone are highly dynamic (Kusick et al., 2019) and so could potentially facilitate this kind of vesicular exchange. Our findings here provide support for this hypothesis, showing that a change in the composition of vesicles apposed to the release machinery accompanies LTP, complementing important recent work identifying nanoscale changes in active zone structure following long-term potentiation (Bell et al., 2014). A possible insight into how the active-zone-associated pool might be modulated comes from our forskolin experiments. We found that activation of the cAMP-PKA pathway—known to interact with proteins associated with vesicle docking such as RIM1a (Castillo et al., 2002, Lonart et al., 2003) and SNAP-25 (Nagy et al., 2004) and playing an established role in LTP induction (Bayazitov et al., 2007, Lonart et al., 2003)—could partially recapitulate the specific changes in fusion site-associated vesicle composition observed with electrically induced LTP. These findings build on important studies that have established key insights into the mechanistic basis for forskolin action in increasing fusion probability and functional pool size at release sites (Midorikawa and Sakaba, 2017, Sakaba and Neher, 2001, Yao and Sakaba, 2010). Moreover, our work focuses attention onto possible downstream targets, for example, SNAP-25, which is phosphorylated by PKA and controls readily releasable pool refilling rates and indirectly, functional pool size (Nagy et al., 2004). Taken together, we suggest that potentiation-driven changes in synaptic vesicle pools might be modular, with those influencing the total recycled pool size distinct from those that regulate the population of vesicles closest to the release machinery, presumably managed by different biochemical pathways. Our study here was limited to an early time point after LTP expression. This might therefore be a transient trace, or alternatively modify functional pool properties in a stable manner that could account for variability of basal fractional pool sizes seen across synaptic populations (Fernandez-Alfonso and Ryan, 2008, Fredj and Burrone, 2009, Harata et al., 2001b, Kim and Ryan, 2010, Li et al., 2005, Micheva and Smith, 2005, Ratnayaka et al., 2012, Rose et al., 2013, Welzel et al., 2011). Characterizing the persistence of the remodeling we observe and its relevance for synaptic strengthening is a key next step.

Our focus was on exploring comparative changes in vesicle pool properties as possible substrates that might encode changes in synaptic efficacy. As such, we did not set out to establish the maximal achievable magnitude of the recycled pool fraction; it is likely that basal pool sizes could have been elevated further by optimizing experimental protocols. Consistent with this, reported mean functional pool sizes vary widely across different studies, from ∼15%–20% (Harata et al., 2001b, Marra et al., 2012) to more than 70% of the total pool (Ikeda and Bekkers, 2009, Rose et al., 2013), reflecting variations in measurement approach, choice of preparation and recruitment paradigms, and with parameters such as the frequency of stimulation and temperature thought to be particularly salient (Denker and Rizzoli, 2010). Moreover, how the LTP modulation we observe maps onto functional changes in circuits occurring *in vivo*, remains to be established. However, it is notable that the few measurements carried out in native intact circuits in behaving animals place the functionally recycled pool size on the low end of the available range (1%–23%) (Denker et al., 2011, Marra et al., 2012), suggesting that there is a large operational range for pool expansion. There is now substantial evidence to support the idea that presynaptic loci can contribute to forms of long-term plasticity in CA3-CA1 circuits (Bayazitov et al., 2007, Bell et al., 2014, Bourne et al., 2013, Chéreau et al., 2017, Emptage et al., 2003, Ma et al., 1999, Malgaroli et al., 1995, Padamsey et al., 2017, Ryan et al., 1996, Tyler et al., 2006, Zakharenko et al., 2001, Zakharenko et al., 2003), adding to evidence for presynaptic LTP mechanisms characterized in other central circuits (e.g., Castillo et al., 1997, Castillo et al., 2002, Lonart et al., 2003, Midorikawa and Sakaba, 2017, Nicoll and Malenka, 1995, Ruiz et al., 2010, Salin et al., 1996, Zhao et al., 2012). Our findings, and those of others, suggest that different parameters of functional vesicle pools, including their size, kinetics, and organization, are important substrates for specifying functional changes, and, perhaps, that different pool subsets might play different roles. The nature of this vesicle pool “code,” however, requires significant further investigation and will be a challenge for future studies, for example taking advantage of readouts that combine function and the latest developments in automated volume-based serial electron microscopy.

## STAR★Methods

### Key Resources Table

**Table:** 

REAGENT or RESOURCE	SOURCE	IDENTIFIER
**Biological Samples**		

Healthy mouse, hippocampal slices	Envigo (Harlan)	N/A

**Chemicals, Peptides, and Recombinant Proteins**		

Ammonium Chloride	Sigma-Aldrich	Cat#254134
6-Cyano-7-nitroquinoxaline-2,3-dione disodium salt (CNQX)	Tocris	Cat#1045
Calcium Chloride	Sigma-Aldrich	Cat#C5080
D(−)-2-Amino-5-phosphonopentanoic acid (AP-5)	Tocris	Cat#0106
Diaminobenzidine (DAB)	Kem-En-Tec	Cat#4170
DMSO	Sigma-Aldrich	Cat#472301
Durcupan resin	Sigma-Aldrich	Cat#44641
FM1-43FX	Invitrogen	Cat#F-35355
Formaldehyde, 16% (wt/vol) solution	Agar Scientific	Cat#AGR1026
Forskolin	Tocris	Cat#1099
Glutaraldehyde, 25% (vol/vol) solution	Agar Scientific	Cat#AGR1312
Glycine	Sigma-Aldrich	Cat#G8898
D-Glucose	Sigma-Aldrich	Cat#G8270
Magnesium chloride	Sigma-Aldrich	Cat#M8266
Osmium tetroxide	TAAB Laboratories	Cat#O021
Potassium chloride	Sigma-Aldrich	Cat#P9333
Potassium ferrocyanide	Sigma-Aldrich	Cat#455989
Sodium cacodylate	Agar Scientific	Cat#AGR1104
Sodium chloride	Sigma-Aldrich	Cat#746398
Sodium hydrogen carbonate	Sigma-Aldrich	Cat#401676
Sodium phosphate, monobasic, monohydrate	Sigma-Aldrich	Cat#S9638
Thiocarbohydrazide	ACROS Organics	Cat#AC207530050
Uranyl acetate	Agar Scientific	Cat#AGR1260A

**Experimental Models: Organisms/Strains**		

Mouse strain c57bl/6	Envigo (Harlan)	N/A

**Software and Algorithms**		

Reconstruct	Fiala, 2005	https://synapseweb.clm.utexas.edu/software-0
MATLAB 2019	MathWorks	https://uk.mathworks.com/
Xara Designer Pro X9	Xara Ltd	https://www.xara.com/us/designer-pro/
Prism 6-8	GraphPad	https://www.graphpad.com/scientific-software/prism/

### Lead Contact and Materials Availability

Further information and requests for resources and reagents should be directed to and will be fulfilled by the Lead Contact, Kevin Staras (k.staras@sussex.ac.uk). This study did not generate new or unique reagents.

### Experimental Model and Subject Details

Experiments were carried out in accordance with the UK-Animal (Scientific Procedures) Act 1986 and satisfied local institutional regulations at the University of Sussex or University of Leicester. The project was given ethical approval by the local Ethical Review Committee (University of Sussex: ARG/1/4; University of Leicester: AWERV_2019_70). Male and female C57BL mice (56-84 days old bred in-house) were used for all experiments.

### Method Details

#### Acute slice preparation

Acute transverse hippocampal slices (300 μm) were prepared using a vibrating blade microtome and maintained in ACSF (artificial cerebrospinal fluid) containing (in mM): 125 NaCl, 2.5 KCl, 25 glucose, 1.25 NaH_2_PO_4_, 26 NaHCO_3_, 1 MgCl_2_, 2 CaCl_2_, 20 μM CNQX, 50 μM AP-5 (bubbled with 95% O_2_ and 5% CO_2_, pH 7.3) (Bayazitov et al., 2007, Staras et al., 2010). Experiments were performed at 28-29°C controlled by a thermostatic inline heater perfusion system. For stimulation, a bipolar tungsten electrode was placed on the Schaffer collaterals and for recording and labeling, a FM1-43FX (Molecular Probes, 20 μM in ACSF)-filled recording electrode (3-5 MΩ) was placed in hippocampal region CA1. The stimulation intensity was set for each experiment so that the evoked fEPSP did not exceed an amplitude/slope of 30%–50% of the maximal stimulation. LTP was induced electrically by three periods of tetanization (each 10 trains of 200 Hz stimulation delivered for 200 ms every 5 s) at 5 min intervals (Bayazitov et al., 2007, Cavuş and Teyler, 1996, Zakharenko et al., 2001, Zakharenko et al., 2003). fEPSP baseline responses were monitored by evoking single electrical stimuli (0.8-1.0 ms duration) at 0.033 Hz before (10 mins) and after induction of LTP. Control experiments used the same timed protocol but without tetanization. At 30 mins post-LTP or post-control, slices were washed into fresh ACSF containing CNQX (20 μM, Tocris) and AP-5 (50 μM, Tocris) to prevent recurrent activity in the network, and FM1-43FX was pressure-applied (∼15 p.s.i. positive pressure) into CA1. The preparation was then left for 3 mins to allow dye to accumulate around the target region before Schaffer collaterals were stimulated at 20 Hz for 30 s (600 APs) to label the recycled pool (Marra et al., 2012, Marra et al., 2014). For some experiments, we added forskolin to the bath solution (50 μM in 0.2% DMSO, Tocris) in place of the LTP induction protocol and perfused continuously until the preparation was subjected to dye-loading. fEPSP baseline responses were established in the same way as for the standard LTP protocol.

#### Photoconversion and ultrastructural investigation

Following completion of dye-loading (3 mins after the end of the stimulation protocol), samples were fixed for 2 mins using rapid microwave fixation (6% gluteraldehyde, 2% formaldehyde in PBS) (Marra et al., 2014). Samples were then transferred to 100 mM glycine (1 h), then rinsed in 100 mM ammonium chloride (1 min) and washed in PBS. For photoconversion, slices were placed on a dedicated photoconversion setup (Dobson et al., 2019) in an oxygen-bubbled diaminobenzidine solution (DAB, 1 mg/ml) and viewed with a 40x 0.8 N.A. water immersion objective. The region of interest, identified from the position of the dye-containing pipette, was illuminated with intense blue light (< 500 nm) for 40 mins. Slices were then washed in PBS followed by ice cold 0.15 M cacodylate buffer containing 2 mM CaCl_2_, and then prepared for electron microscopy following previously described methodology (Deerinck et al., 2010, Rey et al., 2015). In brief, slice samples were placed on ice in a solution containing 3% potassium ferrocyanide in 0.3 M cacodylate buffer containing 4 mM CaCl_2_ mixed with an equal volume of 4% osmium tetroxide (1 h), and then immersed sequentially in filtered warm 1% thiocarbohydrazide solution (20 mins, room temperature), 2% osmium tetroxide (Sigma)(30 mins, room temperature) and 1% uranyl acetate overnight at 4°C. Next, samples were placed in lead aspartate solution in a 60°C oven for 30 mins after which they were successively dehydrated through graded ice-cold alcohols and finally, anhydrous acetone. Samples were then flat-embedded in Durcupan resin and trimmed to the central area in the photoconverted region. The uniform presence of dye-photoconverted vesicles at 2-50 μm below the cut slice surface has previously been demonstrated (Marra et al., 2012, Marra et al., 2014). Samples were sectioned at 60-70 nm and placed on formvar-coated slot grids or 300 mesh. Ultrastructural investigation relied on a Hitachi-7100 or Joel 1400 transmission electron microscope with digital images collected using a 2048 × 2048 CCD camera (Gatan Inc.) or a 5120 × 3840 CMOS camera (EMSIS).

#### Analysis

Images and electron micrographs were analyzed using custom-written MATLAB routines (Mathworks) and Reconstruct (Synapse Web, http://synapseweb.clm.utexas.edu/). Target synapses with PC+ vesicles were randomly selected and each vesicle classified non-blind either as photoconverted (recycled) or non-photoconverted (resting) based on their vesicle lumenal intensity (Darcy et al., 2006, Darcy et al., 2006). Micrographs were aligned and reconstructed using Xara Designer Pro (Xara) and Reconstruct. In previous control experiments where we calibrated our classification of vesicles, we occasionally observed dark vesicular structures that were not consistent with stimulus-evoked labeling (Marra et al., 2012). These likely arise from expected spontaneous recycling events in the period when neurons are incubated in FM-dye or mis-classifications; for example non-equatorial cross-sections through small dense-core vesicles (Sorra et al., 2006). Since it was important that we only included synapses with functional vesicle pools recruited by evoked activity and which therefore also received the potentiating stimulus, we set a lower threshold of pool fraction (> 0.049) for inclusion. Single section analysis was limited to vesicle counts that did not exceed 100 vesicles to ensure uniformity for comparison between groups. Spatial frequency density plots were generated by measuring vesicle coordinate positions and active zone structures using custom-written MATLAB routines. Briefly, representative middle-section electron micrographs were oriented so that the active zone was located at the bottom and the coordinates of each vesicle (either PC+ or PC-), as well as the center point of the active zone, were plotted. Synapses where multiple active zones were observed were not analyzed. Plots of individual synapses (Figure 3A, top) were generated by representing resting and recycled pool vesicle positions on a 10 × 10 grid normalized to the cluster boundaries and color-coded to indicate vesicle density. Note, each pool was normalized to itself such that the total color intensity of all squares summed up to one. For mean density plots (Figures 3A and 3B, bottom), the vesicle positions were calculated to assume lateral symmetry around the midline; non-symmetry is not informative given that synapses are collected from all orientations in the slice. Coordinates for each vesicle were then normalized with respect to the vesicle cluster boundaries and active zone center. These normalized maps for all synapses in one condition were overlaid and used to build the 10 × 10 grid density matrix for PC+ and PC- classes, which was smoothed with a Gaussian filter and color-coded. Note: these plots were used for visual summaries of vesicle positions only; quantitative analysis of vesicle organization was based on raw non-normalized Euclidean distances from each vesicle to its nearest point on the active zone using the original electron micrographs (Figures 3C and 3D). We defined the population of vesicles associated with the active zone as those that lay within 20 nm of the release site membrane and with a clear line of sight. In effect, this population corresponds to the first line of vesicles with access to the active zone. We did not attempt to define a morphologically-docked pool since our images did not allow for unequivocal assessment of tethering structures linked with the AZ and vesicles. For vesicle cluster analysis, we measured the PC+ vesicle fractions in concentric circular regions of interest of increasing size (20 nm radial distance steps) that surrounded each individual PC+ vesicle. All values for these circular bins were expressed as a fraction of PC+ vesicles in the whole synapse. This normalization allowed us to examine changes in clustering independent of the wholesale change in functionally-recycled pool fraction that was observed under different conditions. These cluster index values at each distance were tested for significance against 1 using one sample t tests to establish whether local clustering was significantly different (higher or lower) than the overall (baseline) level of clustering of PC+ vesicles in the terminal. We also examined possible relationships between the degree of clustering and the position in the synapse by dividing the population of vesicles into compartments (rear/side: 300-800 nm, middle: 100-300 nm, front: 35-100 nm, active zone: 0-35 nm) and then running clustering analysis selectively on these regions. To compare these compartments, we first calculated the mean amplitude of the peak cluster (40-240 nm from PC+ vesicle center) for all synapses. 1-way or 2-way ANOVAs were used to test for differences in cluster indexes between compartments within one condition (control or LTP) or between control and LTP respectively.

### Quantification and Statistical Analysis

Statistical tests, N values, what N represents, and other details are included in figure legends or the Results section. Significance was defined as p < 0.05. Statistical comparisons used GraphPad Prism. Datasets were summarized as mean ± SEM (standard error of mean). Two sample comparisons used two-tailed unpaired or paired t tests. Comparisons of multiple datasets used 1-way Kruskal-Wallis ANOVA or 2-way ANOVA. Correlation analysis used Spearman’s rank.

### Data and Code Availability

Datasets supporting the current study have been deposited in a public repository (10.6084/m9.figshare.11567652).

## Supplemental Information

Document S1. Figure S1

Document S2. Article plus Supplemental Information
