# Supplementary material for: Nanoscale Remodeling of Functional Synaptic Vesicle Pools in Hebbian Plasticity
Source: Cell Rep. 2020 Feb 11;30(6):2006–2017.e3. doi: 10.1016/j.celrep.2020.01.051 (PMC7016504; doi:10.1016/j.celrep.2020.01.051)
Supplement: Document S1. Figure S1 [file mmc1.pdf]

Cell Reports, Volume *30*

Supplemental Information

Nanoscale Remodeling of Functional Synaptic Vesicle Pools in Hebbian Plasticity

Stephanie Rey, Vincenzo Marra, Catherine Smith, and Kevin Staras

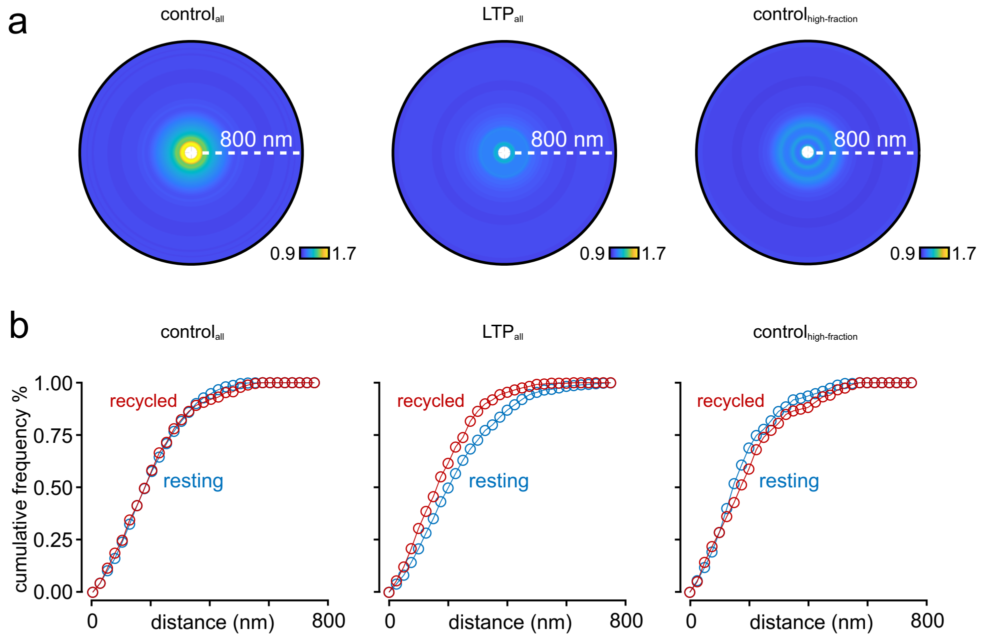

Figure S1. Comparing properties of LTP synapses to the subset of control synapses with comparable pool fractions (control$_{high\text{-}fraction}$). Related to Figures 3 and 4. (a) Mean circular frequency density plots showing relative PC+ clustering, normalized to final recycling fraction for the whole cluster for control$_{all}$ (left), LTP$_{all}$ (middle) and control$_{high\text{-}fraction}$ (right). (b) Cumulative distance plots for control$_{all}$ (left), LTP$_{all}$ (middle) and control$_{high\text{-}fraction}$ (right).
